# An Automatic Image Inpainting Algorithm Based on FCM

**DOI:** 10.1155/2014/201704

**Published:** 2014-01-02

**Authors:** Jiansheng Liu, Hui Liu, Shangping Qiao, Guangxue Yue

**Affiliations:** ^1^College of Science, Jiangxi University of Science and Technology, Ganzhou 341000, China; ^2^Graduate School, Jiangxi University of Science and Technology, Ganzhou 341000, China; ^3^College of Mathematics and Information Engineering, Jiaxing University, Jiaxing 314000, China

## Abstract

There are many existing image inpainting algorithms in which the repaired area should be manually determined by users. Aiming at this drawback of the traditional image inpainting algorithms, this paper proposes an automatic image inpainting algorithm which automatically identifies the repaired area by fuzzy C-mean (FCM) algorithm. FCM algorithm classifies the image pixels into a number of categories according to the similarity principle, making the similar pixels clustering into the same category as possible. According to the provided gray value of the pixels to be inpainted, we calculate the category whose distance is the nearest to the inpainting area and this category is to be inpainting area, and then the inpainting area is restored by the TV model to realize image automatic inpainting.

## 1. Introduction

Inpainting is a trimming process for the defects and cracks of the art, originated from the art field which was originally the heritage restoration of experts on the basis of being faithful to the original. Image inpainting is a technology for restoring the damaged parts of an image by referring to the information from the undamaged parts to make the restored image look “complete”, “continuous” and “natural”.

The terminology “digital image inpainting” was firstly put forward on the international conference in Singapore in 2000. There are many typical image inpainting algorithms proposed by researchers during the past decade. The BSCB [1] model was presented by Bertalmio, Sapiro, Caselles, and Ballester. The TV (total variation) model [2, 3] was proposed by Chan and his team. The CDD (curvature driven diffusions) model [4, 5] was introduced by Chan T. and Shen J. An isotropic diffusion of fast image inpainting was proposed by Oliverira. A model based on RBF was proposed by Zhou et al. [6]. The p-Laplace inpainting model was calculated from p-Laplace operator by Zhang et al. [7–9]. Wang et al. proposed a new spread function to improve the TV model [10], algorithm based on wavelet decomposition was proposed by Zhang and Dai [11] and so forth. However, these models belong to semi-automatic image restoration algorithms that need to manually determine the repaired area. It is a difficult problem for automatically identifying the repaired area of the image and achieving automatically image inpainting function in the field of image inpainting.

This paper attempts to overcome this drawback of the traditional image inpainting techniques to a certain degree. The proposed algorithm utilizes the fuzzy C-means (FCM) clustering algorithm to automatically identify the damaged area and also combines the TV model to realize image automatic inpainting.

## 2. Theoretical Basis of FCM Identify Damaged Areas

### 2.1. Basic Knowledge of the Fuzzy Sets

Fuzzy set theory and fuzzy logic [12] are presented by Zadeh, that is, to use precise methods, formulas, and models to measure and handle phenomena and rules which are vague, incomplete, or incorrect. Membership function is defined as the degree to which an object *x* belongs to a fuzzy set *A*, usually recorded as *u*_*A*_(*x*); the scopes of its argument are the all possible objects which belongs to the set *A* (that all the points of the space where the set *A* in) in the range of [0, 1]; namely, 0 ≤ *u*_*A*_(*x*) ≤ 1. *u*_*A*_(*x*) = 1 means that *x* completely belongs to set *A*, which is equivalent to the traditional set concept *x* ∈ *A*. A membership function defined in the space of *X* = {*x*} is defined as a fuzzy set *A*, or defined as fuzzy subset *A* in the domain of *X* = {*x*}. For the fuzzy set $\underset{\sim }{A}$ which has finite objects *x*_1_, *x*_2_,…, *x*_*n*_ can be expressed as

(1) $$\begin{array}{c} \underset{\sim }{A}=\left\{\left(\mu_{A}\left(x_{i}\right),x_{i}\right)\;|\;x_{i}\in X\right\}. \end{array}$$

Fuzzy set theory has played an important role in many applications, such as fuzzy clustering analysis, fuzzy pattern recognition [13], fuzzy synthetic judgments [14], fuzzy decision and forecast [15, 16], fuzzy programming, fuzzy probability [17], and fuzzy statistics [18]. In the problem of clustering, the clusters generated by clustering can be regarded as fuzzy sets; therefore, the value of membership of each sample point belongs to a cluster that is in the interval [0, 1].

### 2.2. Theory of FCM Algorithm

Fuzzy C-mean clustering (FCM) [19, 20] is a clustering algorithm that uses membership to determine the degree of each data point which belongs to a cluster. In 1973, Bezdek proposed the algorithm that is an improved algorithm of the early hard C-means clustering (HCM). The fuzzy clustering algorithm uses the initialization method to determine several initial clustering centers and then performs a repeated iteration which continuously adjusts and optimizes the clustering centers to achieve the minimal variance within clusters. It is a method based on the principle of least squares using iterative methods to optimize objective function to get the fuzzy partition of the data sets. Its objective function is defined as

(2) $$\begin{array}{c} \min J_{\text{FCM}}\left(U,V\right)=\sum_{k=1}^{n}\;\sum_{i=1}^{c}u_{ik}^{m}d_{ik}^{2}, \end{array}$$

where *X* = {*x*_1_, *x*_2_,…, *x*_*n*_} ∈ *R* ^*n*×*p*^ is a data set that composed of *n* samples of dimension *p*, which is divided it into *C* classes. *x*_*k*_ is a *p*-dimensional vector; *m* ∈ [1, *∞*) is the weighted index; when *m* = 1, the fuzzy C-means clustering is degraded as the classical C-means clustering. Nikhil et al. studies show that the best choice for *m* is in range of [1.5, 2.5] and its ideal value is usually set to *m* = 2; *d*_*ik*_ is generally defined as the Euclidean distance, it means the distance between sample *x*_*k*_ and center *v*_*i*_, that is, *d*_*ik*_ = ||*x*_*k*_−*v*_*i*_||_*A*_, ||·||_*A*_ defines as norm of *A*, usually *A* = 2; that is, *d*_*ik*_ is 2-norm; *V* = {*v*_1_, *v*_2_,…, *v*_*c*_} ∈ *R* ^*c*×*p*^ is the set of *c* cluster centers. *v*_*i*_ is *p*-dimensional vector and denotes the *i*th cluster center; *u*_*ik*_ is the membership of the *k*th pixel that belongs to *i*th class. Therefore, the classification results can be expressed as a *c* × *n*-order fuzzy classification matrix *U*:

(3) $$\begin{array}{c} U=\left[\begin{array}{cccc} u_{11} & u_{12} & \cdots & u_{1n}\\ u_{21} & u_{22} & \cdots & u_{2n}\\ \vdots & \vdots & \ddots & \vdots \\ u_{c1} & u_{c2} & \cdots & u_{cn} \end{array}\right], \end{array}$$

where *u*_*ik*_ need to satisfy the following constraints:

(4) (1) uik∈[0,1], ∀i,k;(2) ∑i=1cuik=1, ∀k;(3) 0<∑i=1cuik<n, ∀i.

FCM is to search the optimum number of (*U*, *V*) for minimizing the *J*_FCM_(*U*, *V*). Due to the constraint ∑_*i*=1_^*c*^ *u*_*ik*_ = 1, it introduces a Lagrange multiplier *λ* to solve the minimum of the following formula:

(5) $$\begin{array}{c} J\left(U,V\right)=\sum_{k=1}^{n}\;\sum_{i=1}^{c}u_{ik}^{m}{||x_{k}-x_{i}||}_{2}^{2}+\lambda \left(\sum_{i=1}^{c}u_{ik}-1\right), \end{array}$$

where

(6) $$\begin{array}{c} u_{ik}=\frac{1}{\sum_{j=1}^{c}{\left[{||x_{k}-v_{i}||}_{2}^{2}/{||x_{k}-v_{j}||}_{2}^{2}\right]}^{1/\left(m-1\right)}},\;\forall i,k,\\ v_{i}=\frac{\sum_{k=1}^{n}u_{ik}^{m}x_{k}}{\sum_{k=1}^{n}u_{ik}^{m}},\;\forall k. \end{array}$$

So the implementation of FCM algorithm is as follows:

**Step 1**

Select *ε* > 0, set the initial clustering centers *V* ^(0)^ = {*v*_1_, *v*_2_,…, *v*_*c*_}, and set the number of iteration *l* = 1.

**Step 2**

The elements of membership matrix *U* ^(*l*)^ are calculated as

(7) $$\begin{array}{c} u_{ik}^{l}={\left\{\sum_{j=1}^{c}{\left[\frac{{||x_{k}-v_{i}||}_{2}^{\left(l\right)}}{{||x_{k}-v_{j}||}_{2}^{\left(l\right)}}\right]}^{2/\left(m-1\right)}\right\}}^{-1},\;\forall i,k. \end{array}$$

If *d*_*kj*_^(*l*)^ = ||*x*_*k*_−*v*_*j*_||_2_^(*l*)^ = 0, then *u*_*kj*_^(*l*)^ = 1, *u*_*kj*_^(*l*)^ = 0, *k* ≠ *j*.

**Step 3**

Calculate the new cluster center set *V* ^(*l*+1)^, whose elements are

(8) $$\begin{array}{c} V^{\left(l+1\right)}=\frac{\sum_{k=1}^{n}\left(u_{ik}^{\left(l\right)}x_{k}\right)x_{k}}{\sum_{k=1}^{n}{\left(u_{ik}^{\left(l\right)}x_{k}\right)}^{m}},\;\forall k. \end{array}$$

**Step 4**

If ||*v* ^(*l*+1)^ − *v* ^(*l*)^|| < *ε*, then stop; otherwise *l* = *l* + 1 and go to Step 2.

## 3. The TV Model

### 3.1. Model Establishment

To establish a normal formula of variation image inpainting model

(9) $$\begin{array}{c} J_{r}\left(u\right)=\int_{\Omega }^{}r\left|\nabla u\right|dx\;dy+\frac{\lambda_{D}}{2}\int_{\Omega }^{}{\left|u-u_{0}\right|}^{2}dx\;dy, \end{array}$$

where

(10) $$\begin{array}{c} \lambda_{D}=\lambda \cdot l_{D}\left(x\right)=\{\begin{array}{cc} 0, & x\in D\\ \lambda , & x\in \Omega \setminus D, \end{array} \end{array}$$

*u*(*x*, *y*) is the image gray function, shorthand for *u*, *u*_0_ is the defect image grayscale function, *D* is the repaired area, *Ω* is the entire image area, *Ω*∖*D* is the known image field, and *λ* is the Lagrange operator, the equation *dδ* = *dx* *dy* is a double integral.

In (9), we have set the energy functional of TV model *r*[|∇*u*|] = |∇*u*|; that is,

(11) $$\begin{array}{c} J_{r}\left(u\right)=\int_{\Omega }^{}\left|\nabla u\right|dx\;dy+\frac{\lambda_{D}}{2}\int_{\Omega }^{}{\left|u-u_{0}\right|}^{2}dx\;dy. \end{array}$$

After calculating out the extreme value of formula (11), we can obtain Euler-Lagrange equation of TV model as follows:

(12) $$\begin{array}{c} -\nabla \cdot \left[\frac{\nabla u}{\left|\nabla u\right|}\right]-\lambda \left(u-u_{0}\right)=0. \end{array}$$

### 3.2. Discretization of the Model

Application of the half-point difference method to calculate the diffusion format of the partial differential equation (12).

**Figure 1:**
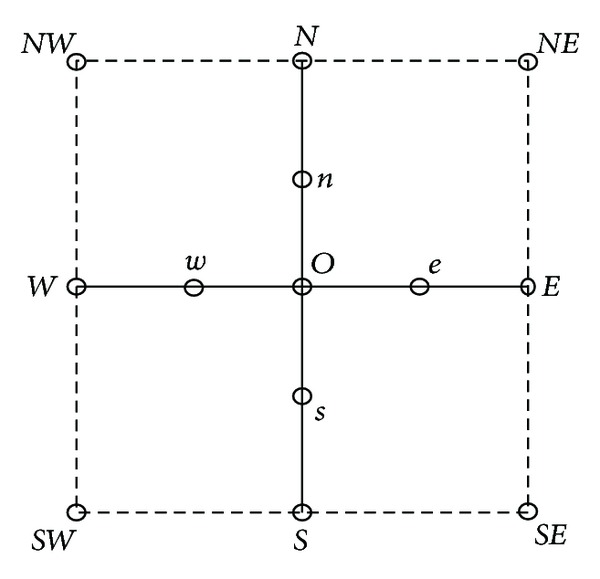
Inpainting pixel points and neighbor fields.

As shown in Figure 1, *O* is the inpainting pixel point, and Λ_0_ = {*N*, *S*, *W*, *E*} means four neighborhood points set of *O*, and Λ = {*n*, *s*, *w*, *e*} means the four half-pixel points. Denote the objective pixel *O* by *u*(*i*, *j*), Λ_0_ = {(*i*, *j* + 1), (*i*, *j* − 1), (*i* − 1, *j*), (*i* + 1, *j*)}. In order to avoid |∇*u*| turning to 0 and take the value of $|\nabla u{|}_{\varepsilon }=\sqrt{\varepsilon^{2}+|\nabla u{|}^{2}}$ or |∇*u*|_*ε*_ = *ε* + |∇*u*|, if *ε* is small enough, it will not affect the performance of TV inpainting model.

Let *v* = (*v* ^1^, *v* ^2^) = ∇*u*/|∇*u*| and take performance about central difference numerical method for divergence,

(13) ∇·v=∂v1∂x+∂v    2∂y≈ve1−vw1h+vn2−vs2h.

The length *h* = 1 and then solve the approach values of *v*_*e*_^1^, *v*_*w*_^1^, *v*_*n*_^2^, and *v*_*s*_^2^:

(14) $$\begin{array}{c} v_{e}^{1}=\frac{1}{{\left|\nabla u_{e}\right|}_{\varepsilon }}{\left[\frac{\partial u}{\partial x}\right]}_{e}\approx \frac{1}{{\left|\nabla u_{e}\right|}_{\varepsilon }}\frac{u_{E}-u_{O}}{h},\\ {\left|\nabla u_{e}\right|}_{\varepsilon }\approx \frac{1}{h}\sqrt{\varepsilon^{2}+{\left(u_{E}-u_{O}\right)}^{2}+{\left(\frac{u_{NE}-u_{SE}+u_{N}-u_{S}}{4}\right)}^{2}}. \end{array}$$

Similarly we can get

(15) $$\begin{array}{c} v_{w}^{1}=\frac{1}{{\left|\nabla u_{w}\right|}_{\varepsilon }}{\left[\frac{\partial u}{\partial x}\right]}_{w}\approx \frac{1}{{\left|\nabla u_{w}\right|}_{\varepsilon }}\frac{u_{W}-u_{O}}{h},\\ v_{n}^{2}=\frac{1}{{\left|\nabla u_{n}\right|}_{\varepsilon }}{\left[\frac{\partial u}{\partial y}\right]}_{n}\approx \frac{1}{{\left|\nabla u_{n}\right|}_{\varepsilon }}\frac{u_{N}-u_{O}}{h},\\ v_{s}^{2}=\frac{1}{{\left|\nabla u_{s}\right|}_{\varepsilon }}{\left[\frac{\partial u}{\partial y}\right]}_{s}\approx \frac{1}{{\left|\nabla u_{s}\right|}_{\varepsilon }}\frac{u_{S}-u_{O}}{h}. \end{array}$$

So

(16) −∇·[∇u|∇u|]=−∇·v=(vw1−ve1)+(vs2−vn2)=∑Q∈ΛO,q∈Λ1|∇uQ|ε[uQ−u(i,j)],

where *Q* ∈ Λ_*O*_, *q* ∈ Λ means that when *Q* is a pixel of Λ_*O*_, then *q* is the corresponding half pixel of Λ; as *Q* take *E*, correspondingly *q* take *e*. So formula (12) can be remarked as

(17) ∑Q∈ΛO,q∈Λ1|∇uQ|ε[uQ−u(i,j)]   +λD(i,j)(u0(i,j)−u(i,j))=0.

So,

(18) u(i,j)=∑Q∈ΛO,q∈Λ(1/|∇uQ|ε)∑Q∈ΛO,q∈Λ(1/|∇uQ|ε)+λD(i,j)uQ+λD(i,j)∑Q∈ΛO,q∈Λ(1/|∇uQ|ε)+λD(i,j)u0(i,j),

where

(19) $$\begin{array}{c} \omega_{q}=\frac{1}{{\left|\nabla u_{Q}\right|}_{\varepsilon }},\;\;h_{Q}=\frac{\omega_{q}}{\lambda_{D}\left(O\right)+\sum_{Q\in \Lambda_{O},q\in \Lambda }\omega_{q}},\\ h_{O}=\frac{\lambda_{D}\left(O\right)}{\lambda_{D}\left(O\right)+\sum_{Q\in \Lambda_{O},q\in \Lambda }\omega_{q}}. \end{array}$$

So formula (18) can be remarked as:

(20) $$\begin{array}{c} u\left(i,j\right)=\sum_{Q\in \Lambda_{O},q\in \Lambda }h_{Q}u_{Q}+h_{O}u_{O}\left(i,j\right), \end{array}$$

where ∑_*Q*∈Λ_*O*_,*q*∈Λ_ *h*_*Q*_ + *h*_*O*_ = 1; apparently, the inpainting objective pixel value *u*(*i*, *j*) adds the weight *h*_*Q*_ to be repaired through neighbor field point *u*_*Q*_.

Use Gauss-Jacobi iteration method to search the optimum:

(21) $$\begin{array}{c} u^{\left(n\right)}\left(i,j\right)=\sum_{Q\in \Lambda_{O},q\in \Lambda }h_{Q}^{\left(n-1\right)}u_{Q}^{\left(n-1\right)}+h_{O}^{\left(n-1\right)}u_{O}\left(i,j\right), \end{array}$$

where *n* is the number of iterations. According to the discretization process of TV model, the process of TV inpainting model mainly is shown in Algorithm 1.

## 4. Computer Experiment

### 4.1. FCM Experimental Analysis

In the FCM experiment in this paper given gray values of the pixel which in the repaired areas, we can calculate the Euclidean distance of it with each clustering center concluded by formula (8), the cluster which has the minimum distance is the repaired area *D*.

In this experiment, it is crucial to select the appropriate values of these parameters, namely, the number of image clustering (*C* value), the initial value of the clustering center (*V*), *C*, and the clustering center initial value selected which directly influence whether the defect area can be accurately identified. Generally apply the gray value distribution of the gray histogram of the image to be repaired to determine the value of *C* and *V*.

We discussed the experimental results of FCM to identify scratches, text, and big block inpainting areas

#### 4.1.1. Scratch Inpainting

**Figure 2:**
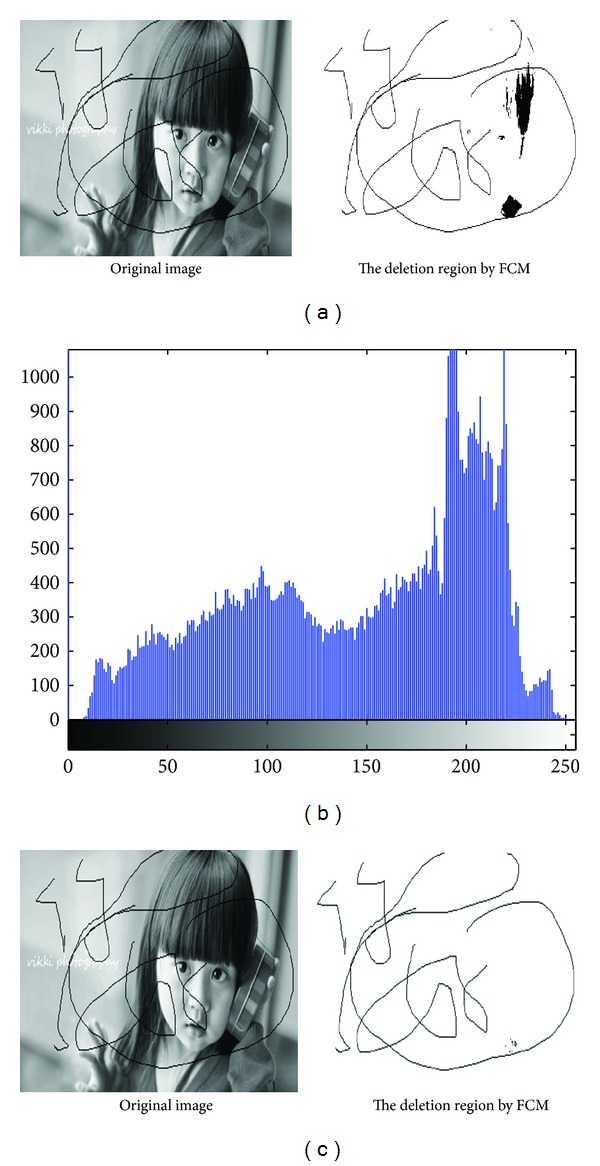
(a) Identification effect of areas to be repaired with *C* = 6. (b) The gray histogram of image to be repaired. (c) Identification effect of areas to be repaired with *C* = 8.

In Figure 2(a) there is identification effect of areas to be repaired; Figure 2(b) is gray histogram of Figure 2(a).

As Table 1 has shown in the experiment, we set *C* = 6; to initialize clustering centers *V* = [0, 50, 100, 125, 175, 255] and update the clustering centers, obtained clustering center 1 is 21.6442, and the clustering center 2 is 69.3399, the clustering center 3 is 108.8676, the clustering center 4 is 154.7014, the clustering center 5 is 192.2138, and the clustering center 6 is 217.2253. Apparently the area to be repaired in cluster 1 identifying the scratches of the repaired area was shown in Figure 2(a), the pixel in the image to be repaired where it close to black gray value will impact identification of the repaired areas from the figure.

When set *C* = 8, identification effect of areas to be repaired was shown in Figure 2(c).

Apparently, the effect of Figure 2(c) is better than that of Figure 2(a); at this time, we initialize the clustering center *V* = [0, 20, 50, 75, 100, 125, 175, 255] and the clustering center 1 is 9.6953, the clustering center 2 is 45.918, the clustering center 3 is 78.5321, the clustering center 4 is 107.6517, the clustering center 5 is 139.0719, the clustering center 6 is 169.5539, the clustering center 7 is 196.3286, the clustering center 8 is 218.8029. Area to be repaired which has been in Cluster 1, as shown in Tables 1 and 2, the cluster center value of 1 is closer to the color of the area to be repaired (gray value is 0) than the cluster center value of 1 when *C* = 6. Therefore, for the greater value of *C*, the identification is more accurate.

#### 4.1.2. Text Inpainting

**Figure 3:**
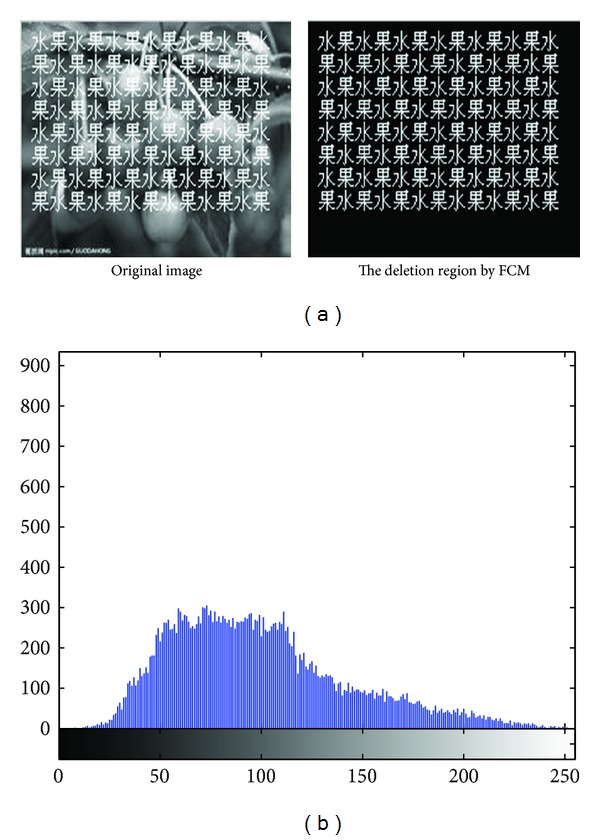
(a) Identification effect of areas to be repaired with *C* = 5. (b) The gray histogram of image to be repaired.

In Figure 3(a), there is the identification result of the areas to be repaired; Figure 3(b) is gray histogram of Figure 3(a).

As Table 3 shows, in the experiment, we set *C* = 5 initialize clustering center *V* = [0,75,150,200,255], obtained the clustering center 1 is 52.0827, the clustering center 2 is 85.3504, the clustering center 3 is 119.696, the clustering center 4 is 171.9693, and the clustering center 5 is 254.6589. Apparently, the area to be repaired in cluster 5 identifying the scratches of the repaired area was shown in Figure 3(a).

#### 4.1.3. The Big Block Area Inpainting

**Figure 4:**
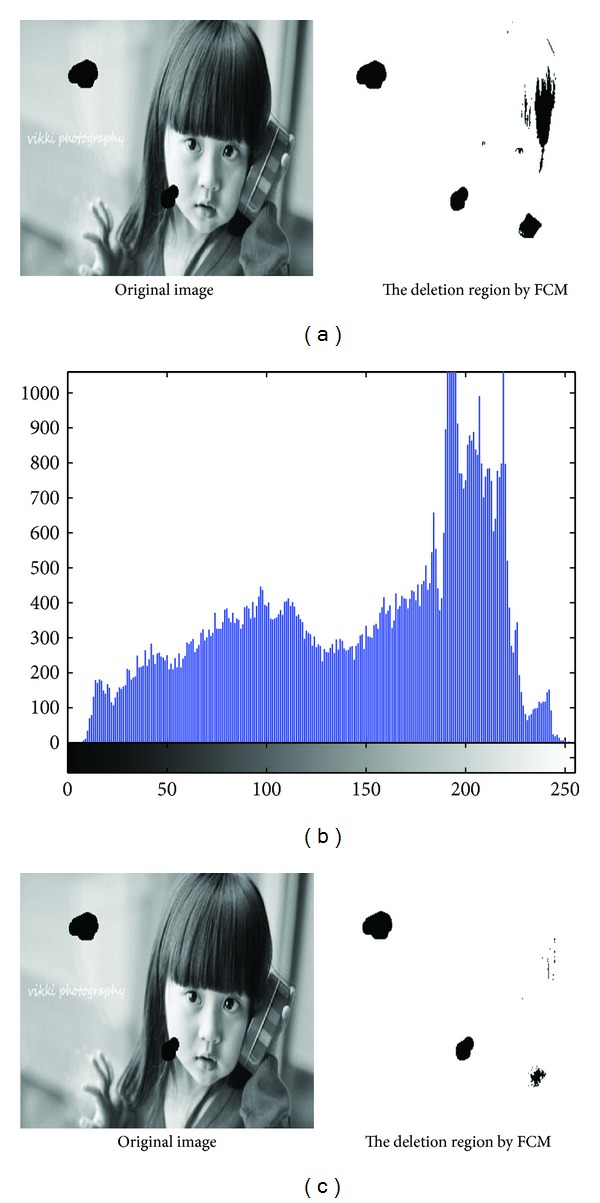
(a) Identification effect of areas to be repaired with *C* = 7. (b) The gray histogram of image to be repaired. (c) Identification effect of areas to be repaired with *C* = 10.

In Figure 4(a), there is the identification result of areas to be repaired; Figure 4(b) presents gray histogram of Figure 4(a).

As Table 4 has shown, in the experiment, we set *C* = 7, to initialize clustering center *V* = [10,20,50,100,125,175,255], update the clustering center, finally obtained the clustering center 1 is 22.732, the clustering center 2 is 60.23, the clustering center 3 is 93.2463, the clustering center 4 is 124.5873, the clustering center 5 is 162.8123, the clustering center 6 is 194.4558, and the clustering center 7 is 217.9387. Apparently, area to be repaired in cluster 1 identifying the scratches of the repaired area was shown in Figure 4(a); the pixels in the image to be repaired nearly black will be misclassified as noise, so if the grayscale values of the area to be repaired are similar to the gray values of the known area, they will be misclassified.

When we set *C* = 10, the extracted area to be repaired was shown in Figure 4(c).

Apparently, the result of Figure 4(c) is better than Figure 4(a); at this time, to initialize clustering center *V* = [0,10,20,30,40,50,100,125,175,255], update the clustering center, finally obtained the clustering center 1 is 12.2282, the clustering center 2 is 39.6536, the clustering center 3 is 63.6337, the clustering center 4 is 85.1273, the clustering center 5 is 106.6592, the clustering center 6 is 129.8529, the clustering center 7 is 156.3771, the clustering center 8 is 179.4131, the clustering center 9 is 198.9847, and the clustering center 10 is 219.6205. At this time, Area to be repaired which has been in Cluster 1, as shown in Tables 4 and 5, the cluster center value of 1 is closer to the color of the area to be repaired (gray value is 0) than the cluster center value of 1 when *C* = 7. Therefore, the greater value of *C* and the initial value of clustering center are more appropriate, and the more accurate identification results can be obtained.

### 4.2. Realization of the Image Automatic Inpainting

Let *λ* = 0, *ε* = 0.0001 with improved signal-to-noise (ISNR) as the objective metric standard of the quantity of the inpainting image, the definition of ISNR as

(22) $$\begin{array}{c} \text{ISNR}=10\cdot \log_{\text{10}}\left\{\frac{\sum_{x=1}^{M}\sum_{y=1}^{N}{\left[\hat{I}\left(x,y\right)-I^{0}\left(x,y\right)\right]}^{2}}{\sum_{x=1}^{}\sum_{y=1}^{}{\left[\hat{I}\left(x,y\right)-I^{0}\left(x,y\right)\right]}^{2}}\right\}, \end{array}$$

where $\hat{I}(x,y)$ is the original gray image, *I* ^0^(*x*, *y*) is the damaged image, and *I*(*x*, *y*) is the recovered image.

Combining TV model inpainting algorithm with FCM identifying the damaged areas, we have discussed the automatic inpainting effect of scratches and big block damaged areas as follows.

#### 4.2.1. Scratch Inpainting

In the following experiments, *n* is the number of iteration, *T* is the inpainting time, and ISNR is improved signal-to-noise.

**Figure 5:**
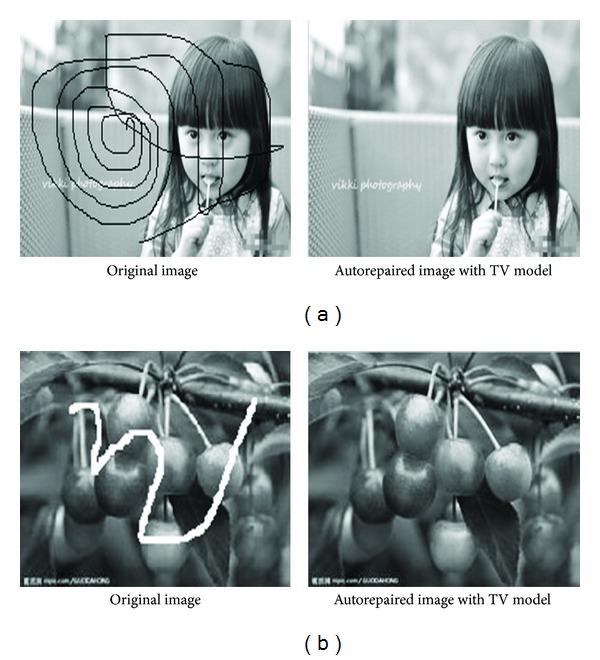
(a) *n* = 50, *T* = 0.0832, ISNR = 60.648. (b) *n* = 50, *T* = 0.0685, ISNR = 53.0085.

Figures 5(a) and 5(b) show that the automatic inpainting model has good inpainting effect on fine scratches and coarse scratches and can accurately identify the different kinds of image noise.  Figure 5(a) is black noise, and Figure 5(b) is white noise.

#### 4.2.2. The Big Block Area Inpainting

**Figure 6:**
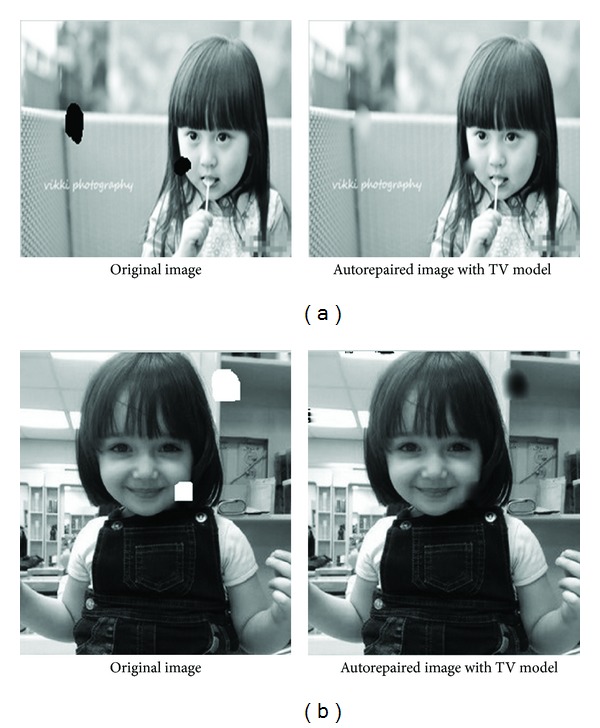
(a) *n* = 50, *T* = 0.0598, ISNR = 35.5889. (b) *n* = 50, *T* = 0.1054, ISNR = 10.0905.

Comparing Figures 5 and 6, it shows that the automatic image inpainting model may generate blurred edges for big block damaged area, not as good as small inpainting areas.

## 5. Conclusions

FCM clustering is an unsupervised clustering technique applied to classify images into clusters with similar properties. It utilizes the distance between pixels and cluster centers in the repaired area to compute the membership function. Experiments showed that the inpainting effect of automatic image inpainting model was decided by the inpainting model, identifying the repaired area that depends on FCM algorithm. When the selected clustering number *C* and the selected clustering center initial value are appropriate, FCM can accurately identify the repaired area. As combined with the inpainting model, we can realize automatic digital image inpainting function.

## Figures and Tables

**Algorithm 1:**
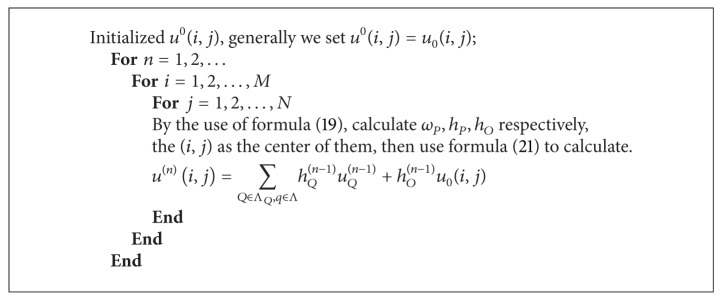


**Table 1:** Clustering centers table.

Initial and obtained cluster centers		
	Initial clustering centers	Obtained clustering centers
Clustering center 1	0	21.6442
Clustering center 2	50	69.3399
Clustering center 3	100	108.8676
Clustering center 4	125	154.7014
Clustering center 5	175	192.2138
Clustering center 6	255	217.2253

**Table 2:** Clustering centers table.

Initial and obtained clustering center		
	Initial clustering centers	Obtained clustering centers
Clustering center 1	0	9.6953
Clustering center 2	20	45.918
Clustering center 3	50	78.5321
Clustering center 4	75	107.6517
Clustering center 5	100	139.0719
Clustering center 6	125	169.5539
Clustering center 7	175	196.3286
Clustering center 8	255	218.8029

**Table 3:** Clustering centers table.

Initial and obtained clustering center		
	Initial clustering centers	Obtained clustering centers
Clustering center 1	0	52.0827
Clustering center 2	75	85.3504
Clustering center 3	150	119.696
Clustering center 4	200	171.9693
Clustering center 5	255	254.6589

**Table 4:** Clustering centers table.

Initial and obtained clustering centers		
	Initial clustering centers	Obtained clustering centers
Clustering center 1	10	22.732
Clustering center 2	20	60.23
Clustering center 3	50	93.2463
Clustering center 4	100	124.5873
Clustering center 5	125	162.8123
Clustering center 6	175	194.4558
Clustering center 7	255	217.9387

**Table 5:** Cluster center table.

Initial and obtained clustering centers		
	Initial clustering centers	Obtained clustering centers
Clustering center 1	0	12.2282
Clustering center 2	10	39.6536
Clustering center 3	20	63.6337
Clustering center 4	30	85.1273
Clustering center 5	40	106.6592
Clustering center 6	50	129.8529
Clustering center 7	100	156.3771
Clustering center 8	125	179.4131
Clustering center 9	175	198.9847
Clustering center 10	255	219.6205

## References

[1] Zeng C, Wang M (2009). Fast convergent image inpainting based on the BSCB model. *Journal of Algorithms and Computational Technology*.

[2] Tai XC, Osher S, Holm R (2007). Image inpainting using a TV-Stokes equation. *Image Processing Based on Partial Differential Equations*.

[3] Osher S, Burger M, Goldfarb D, Xu J, Yin W (2005). An iterative regularization method for total variation-based image restoration. *Multiscale Modeling and Simulation*.

[4] Brito-Loeza C, Chen K (2008). Multigrid method for a modified curvature driven diffusion model for image inpainting. *Journal of Computational Mathematics*.

[5] Liu J, Li M, He F (2012). A novel inpainting model for partial differential equation based on curvature function. *Journal of Multimedia*.

[6] Zhou T, Tang F, Wang J, Wang Z, Peng Q (2004). Digital image inpainting with radial basis functions. *Journal of Image and Graphics*.

[7] Zhang H The Research and Application of digital image inpainting tehchnology.

[8] Zhang H, Peng Q An image repair method based on p-Laplace operator.

[9] Zhang H, Peng Q, Wu YD (2006). Digital image inpainting algorithm for damaged images based on nonlinear anisotropic diffusion. *Journal of Computer-Aided Design and Computer Graphics*.

[10] Wang T, Wang J, Zhang W (2013). Improved method of total variation image inpainting. *Journal of Computer Systems and Applications*.

[11] Zhang H, Dai S (2012). Image inpainting based on wavelet decomposition. *Procedia Engineering*.

[12] Jun H, Guoyin W (2010). Covering based generalized rough fuzzy set model. *Journal of Software*.

[13] Xu M, Duffield C, Ma J (2011). Performance of mid-project reviews (MPRs): quantification based on fuzzy recognition. *Built Environment Project and Asset Management*.

[14] Lu H, Yuan H, Liu H (2012). Improve max-min algorithm of fuzzy synthetic. *Journal of PLA University of Science and Technology (Natural Science Edition)*.

[15] Cavallo A, Di Nardo A, de Maria G, Di Natale M (2013). Automaticed fuzzy decision and control system for reservoir management. *Journal of Water Supply: Research and Technology—AQUA*.

[16] Rakityanskaya AB, Rotshtein AP (2005). Fuzzy forecast model with genetic-neural tuning. *Journal of Computer and Systems Sciences International*.

[17] Iskander MG (2012). An approach for linear programming under randomness and fuzziness: a case of discrete random variables with fuzzy probabilities. *International Journal of Operational Research*.

[18] Turanli N (2013). Using fuzzy statistics to determine mathematics attitude and anxiety. *Middle-East Journal of Scientific Research*.

[19] Xu Z, Wu J (2010). Intuitionistic fuzzy C-means clustering algorithms. *Journal of Systems Engineering and Electronics*.

[20] Miyamoto S (2008). *Algorithms for Fuzzy Clustering: Methods in C-Means Clustering with Applications*.

